# *In Silico* Analysis of Relationship between Proteins from Plastid Genome of Red Alga *Palmaria* sp. (Japan) and Angiotensin I Converting Enzyme Inhibitory Peptides

**DOI:** 10.3390/md17030190

**Published:** 2019-03-25

**Authors:** Yuya Kumagai, Yoshikatsu Miyabe, Tomoyuki Takeda, Kohsuke Adachi, Hajime Yasui, Hideki Kishimura

**Affiliations:** 1 Laboratory of Marine Chemical Resource Development, Faculty of Fisheries Sciences, Hokkaido University, Hakodate, Hokkaido 041-8611, Japan; yuyakumagai@fish.hokudai.ac.jp; 2 Chair of Marine Chemical Resource Development, Graduate School of Fisheries Sciences, Hokkaido University, Hakodate, Hokkaido 041-8611, Japan; yoshikatsu_miyabe@aomori-itc.or.jp (Y.M.); sekaiha3146@ezweb.ne.jp (T.T.); 3 Laboratory of Aquatic Product Utilization, Graduate School of Agriculture, Kochi University, Monobeotsu 200, Nankoku, Kochi 783-8502, Japan; kohsukeadachi@kochi-u.ac.jp; 4 Laboratory of Humans and the Ocean, Faculty of Fisheries Sciences, Hokkaido University, Hakodate, Hokkaido 041-8611, Japan; hagime@fish.hokudai.ac.jp

**Keywords:** dulse, *Palmaria* sp. (Japan), ACE inhibitory peptide, plastid genome

## Abstract

Plastid proteins are one of the main components in red algae. In order to clarify the angiotensin I converting enzyme (ACE) inhibitory peptides from red alga *Palmaria* sp. (Japan), we determined the plastid genome sequence. The genome possesses 205 protein coding genes, which were classified as genetic systems, ribosomal proteins, photosystems, adenosine triphosphate (ATP) synthesis, metabolism, transport, or unknown. After comparing ACE inhibitory peptides between protein sequences and a database, photosystems (177 ACE inhibitory peptides) were found to be the major source of ACE inhibitory peptides (total of 751). Photosystems consist of phycobilisomes, photosystem I, photosystem II, cytochrome complex, and a redox system. Among them, photosystem I (53) and II (51) were the major source of ACE inhibitory peptides. We found that the amino acid sequence of apcE (14) in phycobilisomes, psaA (18) and psaB (13) in photosystem I, and psbB (11) and psbC (10) in photosystem II covered a majority of bioactive peptide sequences. These results are useful for evaluating the bioactive peptides from red algae.

## 1. Introduction

Marine algae contain proteins, lipids, carbohydrates, vitamins, and minerals as nutrition. The amount of these elements vary depending on season and the area of production [1,2]. Seaweed can be used as a source of polysaccharides, such as alginate, carrageenan, and agar [3,4]. Asia has a long tradition of consuming seaweed and seaweed has recently become considered a health food worldwide [5].

Among seaweeds, red algae contain a high amount of protein compared to green and brown algae [1,6]. The amount of protein varies according to environmental conditions and ranges from 7% to 30% [1,7]. The main components of protein in red algae are phycobiliproteins and ribulose-1,5-bisphosphate carboxylase/oxygenase (Rubisco). Phycobiliproteins form the complex structure of phycobilisomes, with phycobiliproteins and chromophores that capture light energy for photosynthesis [8]. The chromophores are used as the antioxidant materials in this process [9,10]. The proteinase hydrolysate of the rod-shaped protein of phycobiliproteins and Rubisco has different bioactivities, such as inhibition of both angiotensin I converting enzyme (ACE) and dipeptidyl peptidase IV (DPP IV) [11,12,13,14,15,16,17,18,19,20,21,22]. Bioactive peptides have been reported in various protein sources [18,23]. The typical strategy for the identification of peptides includes a series of steps: peptide production using proteinases, preparation, inhibitory activity measurement, identification of peptide sequences, and confirmation of the activity using a synthesized peptide [12,13,14,24]. Some studies have confirmed this peptide inhibitory activity in animal experiments [24]. This method is useful for the identification of novel and major peptide sequences in samples. However, it is difficult to identify a small amount of peptide that has strong activity in a sample, as the peptide contributes its activity to the whole hydrolysate sample. The data for peptide sequences and inhibitory concentration (IC_50_) can be found in a database (http://www.uwm.edu.pl/biochemia/index.php/pl/biopep). These data were obtained from various protein sources. It has been speculated that the same value of biological activity would be expressed by peptides obtained from different sources. Therefore, it was hypothesized that finding the peptide sequences in the protein sequences from genomes would unveil functional peptides from natural sources.

In this study, we determined the complete plastid genome sequence of *Palmaria* sp. (Japan) and annotated protein coding genes (PCGs), which are the main source of proteins in red algae. To discover functional peptides, the relationship between protein sequences in the plastid and the database was evaluated. 

## 2. Results and Discussion

### 2.1. General Features of Palmaria sp. (Japan) Plastid Genomes

**Figure 1:**
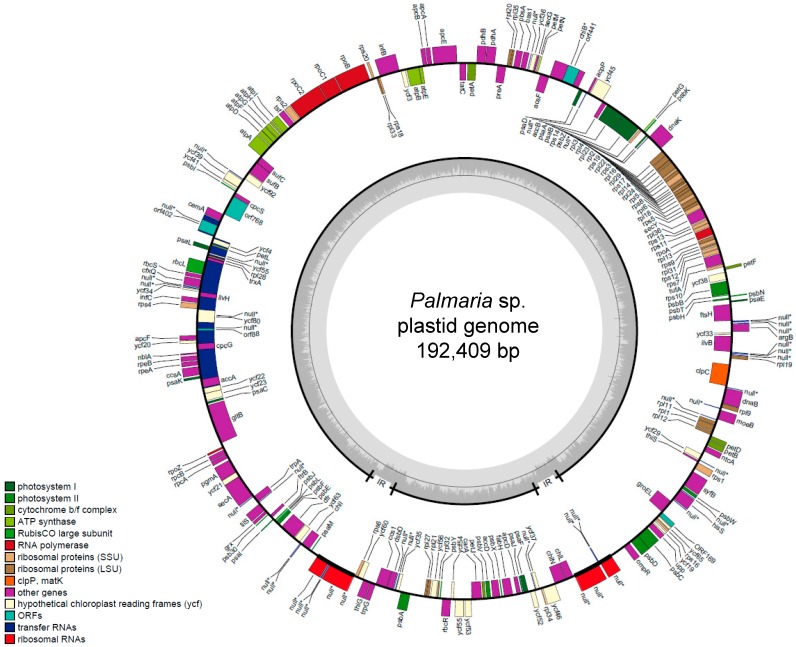
The plastid genome map of *Palmaria* sp. (Japan).

The complete plastid genomes of *Palmaria* sp. (Japan) were determined using next-generation sequencing (NGS) methods. The contigs coding plastid were assembled using BLASTn before we obtained the draft circular plastid genome. The genes in the plastid were annotated manually and the gap or deletion in PCGs were confirmed using PCR amplification followed by Sanger sequencing using specific primers (Table S1). As a result, a total of 192,409 nt of the plastid genome was sequenced (Figure 1). The average coverage for the plastid genomes was 630×. The genome contained 205 PCGs (Table 1). The plastid sequence was deposited in DNA Data Bank of Japan (DDBJ) as AB807662. 

When comparing the architecture of plastid genomes between *Palmaria* sp. (Japan) and the related species, the plastid genome was most similar to that of *Palmaria palmata*. This similarity was namely in terms of two introns, 205 PCGs, 33 tRNAs, and two copies of the ribosomal RNA operon (Table 2). Although the genes were completely conserved, *Palmaria* sp. (Japan) had a small total number of nt (192,409) and high GC content (34.6%) compared to *P. palmata,* which had 192,960 nt and 33.9% GC content.

### 2.2. Comparison of Amino Acid (AA) Composition between Palmaria sp. (Japan) Plastid Proteins and Proximate AA in P. palmata

The AA compositions of marine algae have been studied for a long period of time [30]. The AA compositions, which are an important source of protein, differ between algae species. This suggests that the differences may reflect the composition of the final product. Therefore, the AA composition of plastid proteins and the real composition in *P. palmata* were compared (Table 3). The AA composition was quite similar, except for aspartic acid and glycine in real protein, and isoleucine and leucine in real AA and protein. Mai et al. reported on the AA composition in various types of seaweed, and showed that the amount of aspartic acid and glycine was mostly stable in seaweed [6,31]. Therefore, we focused on the amounts of isoleucine and leucine. The amounts of isoleucine (9.0%) and leucine (10.1%) in plastid proteins was higher than the true AA and protein (isoleucine 5.3% and 3.7%; leucine 7.8% and 7.1%). The proportions found in plastid proteins showed that the proteins were equally expressed. Focusing on the classification of protein function, the amount of isoleucine and leucine in ribosomal protein (8.7% and 8.7%) and isoleucine in phycobilisomes (7.5%) was low. Therefore, considering the fact that ribosomal protein and phycobilisomes proteins are the main red algae proteins, the percentage of AA in the real seaweed would be close to the composition of plastid proteins. Although there is currently no information on nuclear and mitochondrial genomes, it would be expected that the proteins from these genomes would contain low amounts of isoleucine and leucine. 

### 2.3. ACE Inhibitory Peptides in Plastid

ACE inhibitory peptides have been found in red algae proteins, which are namely the rod-like proteins of phycobilisomes and Rubisco, because these are the major components of soluble red algae proteins [32]. The increase in accessibility to the protein was previously studied [5,33]. However, 205 PCGs exist in the *Palmaria* sp. (Japan) plastid genome, which indicates that the insoluble or membrane proteins have potential as a source of bioactive peptides. Therefore, we screened the plastid proteins to confirm the possibility of using them as bioactive peptides. ACE inhibitory tripeptides with IC_50_ less than 20 μM were extracted from the biopep-uwm database, and a total of 89 peptides were selected. Although di-, tetra-, or longer peptides with ACE inhibitory activity were deposited in the database, we employed the tripeptide database to reduce overestimation. A large proportion of these peptides consisted of proline (34 peptides) or tyrosine (20 peptides) at the C-terminus. After comparing the plastid proteins and the peptide sequences, a total of 751 ACE inhibitory peptides were found (Table 4). When the peptide sources were classified according to protein function, photosystems contained the highest number with 177 peptides, followed by metabolism (176) and ribosomal proteins (128). The smallest number of peptides were involved in ATP synthesis (28), according to functional classification. This was due to a small proportion of total AAs involved in ATP synthesis.

### 2.4. ACE Inhibitory Peptides in Photosystems

It has been reported that the proteins from photosystems are the major components of soluble proteins, with these proteins containing various types of bioactive peptides [23]. Photosystems contain a large number of ACE inhibitory peptides (Table 4). Therefore, we investigated the peptides in photosystems. The function of photosystem proteins was classified into phycobilisomes, photosystem I, photosystem II, cytochrome complex, and redox system. Among them, photosystem I had the highest number with 53 peptides, followed by photosystem II (51), and phycobilisomes (42) (Table 5). The ratio of the number of peptides to the total AA (peptide/AA (%)) was high in photosystem I (2.00%) and photosystem II (1.98%) compared with photosystems (1.59%) and plastid (1.49%). After this, we focused on the number of ACE inhibitory peptides in proteins. We found that the proteins of apcE, psaA, psaB, psbA, psbB, and psbC possessed a large number of the peptides (Table 6). The photosystem proteinspsaA, psaB, psbA, psbB, and psbC are the components of the integral membrane proteins in photosystem I and II, which are not easily obtained through water extraction as soluble proteins. Most ACE inhibitory peptides from red algae were from soluble proteins, that is, from the rod-like proteins of phycobilisomes and Rubisco. These data are useful for finding novel bioactive peptides from red algae proteins.

### 2.5. Comparison of ACE Inhibitory Peptides in Palmaria sp. (Japan) and P. palmata

The plastid genomes of *Palmaria* sp. (Japan) and *P*. *palmata* were similar, and the number of PCGs was the same (205). To clarify the differences in ACE inhibitory peptides between *Palmaria* sp. (Japan) and *P*. *palmata*, the ACE inhibitory peptides were compared (Table 7). A total of 742 peptides were found in *P*. *palmata*, which was less than that found in *Palmaria* sp. (751). The difference was due to an unknown protein that had 80 peptides in *Palmaria* sp. and 72 peptides in *P*. *palmata*. Although the number of peptides among the other protein functional groups was almost the same, the peptide sequences differed between these groups (Table 4; Table 7). These data are useful for selecting peptide producing proteinases. 

## 3. Materials and Methods

### 3.1. Plastid Genome Construction

*Palmaria* sp. was collected from Usujiri, Japan in February 2012. Genomic DNA was extracted using the hexadecyltrimethylammonium bromide (CTAB) method [34]. The genome sequence data were generated using the GS Junior Titanium Series system (Roche). After this, the DNA library was subjected to emulsion PCR (emPCR) using the emPCR Reagents kit (Lib-A) (Roche) according to the manufacturer’s protocol. After emPCR, DNA beads were enriched and placed on a picotiter plate (Roche) before we ran generation sequencing on this DNA using the GS Junior equipment (Roche). The contigs coding plastids were assembled with BLASTn using the red algal *P. palmata* plastid genes as a reference (NC_031147.1). After the reassembly, a circular plastid genome was obtained. The genes coding proteins were manually annotated using RNAmmer v1.2 server (http://www.cbs.dtu.dk/services/RNAmmer/), tRNAscan-SE 2.0 (http://lowelab.ucsc.edu/tRNAscan-SE/), ORF finder (https://www.ncbi.nlm.nih.gov/orffinder/). A gap in genes was confirmed by PCR amplification and Sanger sequencing (Table S1). The annotated plastid genomes were visualized using OrganellarGenomeDraw v1.2 [35]. 

### 3.2. Collection of ACE Inhibitory Peptides and Comparison with Plastid Proteins

ACE inhibitory peptides were obtained from the biopep-uwm database (http://www.uwm.edu.pl/biochemia/index.php/pl/biopep) on 28 January 2019. From the database, tripeptides with IC_50_ less than 20 μM were selected. The peptide sequences in plastid proteins were manually annotated.

## 4. Conclusions

We determined the complete plastid genome sequence of the red alga *Palmaria* sp. (Japan) and annotated 205 PCGs. Comparing the plastid protein sequences and ACE inhibitory peptide sequences to a database, a large part of the peptide sequences was classified into photosystems (177) and metabolism (176). Among the photosystems, the proteins from apcE, psaA, psaB, psbA, psbB, and psbC possessed a large number of the peptides. Comparing protein sequences between *Palmaria* sp. (Japan) and *P*. *palmata*, the number of ACE inhibitory peptides was similar, although they had a different composition of peptides. We previously prepared ACE inhibitory peptides from water-extracted dulse protein as thermolysin hydrolysate [15]. The peptide sequences identified were mainly from phycobiliproteins. We therefore could not identify peptides from membrane proteins such as photosystem I and II. *In silico* analysis showed both the potential of membrane proteins for ACE inhibitory peptides and the characteristic C-terminal structure of ACE inhibitory peptides. Digestive enzymes such as pepsin (Aps, Glu, Leu, Phe, Trp, and Tyr), chymotrypsin (Phe, Trp, and Tyr), elastase (Ala, Gly, Ile, Leu, Ser, and Val), and prolyl endopeptidase (Pro) hydrolyzed the C-terminus of proteins, and would produce ACE inhibitory peptides. We expected that peptides from membrane proteins, which were not identified in in vitro experiments, would play a role in the inhibition of high blood pressure. In addition to ACE inhibitory activity, DPP IV inhibitory peptides were also identified in red algae protein hydrolysates, and *in silico* analysis would apply for finding novel bioactive peptides from red algae proteins. 

## Figures and Tables

**Table 1:** Protein coding genes (PCGs) in *Palmaria* sp. (Japan).

Classification		No.	Gene							
**Genetic System**	Maintenance	2	*dnaB*	*rne*						
	RNA polymerase	5	*rpoA*	*rpoB*	*rpoC1*	*rpoC2*	*rpoZ*			
	Transcription factors	4	*ntcA*	*ompR*	*rbcR*	*ycf29*				
	Translation	4	*infB*	*infC*	*tsf*	*tufA*				
**Ribosomal Proteins**	Large subunit	28	*rpl1*	*rpl2*	*rpl3*	*rpl4*	*rpl5*	*rpl6*	*rpl9*	*rpl11*
			*rpl12*	*rpl13*	*rpl14*	*rpl16*	*rpl18*	*rpl19*	*rpl20*	*rpl21*
			*rpl22*	*rpl23*	*rpl24*	*rpl27*	*rpl28*	*rpl29*	*rpl31*	*rpl32*
			*rpl33*	*rpl34*	*rpl35*	*rpl36*				
	Small subunit	19	*rps1*	*rps2*	*rps3*	*rps4*	*rps5*	*rps6*	*rps7*	*rps8*
			*rps9*	*rps10*	*rps11*	*rps12*	*rps13*	*rps14*	*rps16*	*rps17*
			*rps18*	*rps19*	*rps20*					
	tRNA processing	1	*tilS*							
	Protein quality control	4	*clpC*	*dnaK*	*ftsH*	*groEL*				
**Photosystems**	Phycobilisomes	12	*apcA*	*apcB*	*apcD*	*apcE*	*apcF*	*rpcA*	*rpcB*	*cpcG*
			*cpcS*	*rpeA*	*rpeB*	*nblA*				
	Photosystem I	13	*psaA*	*psaB*	*psaC*	*psaD*	*psaE*	*psaF*	*psaI*	*psaJ*
			*psaK*	*psaL*	*psaM*	*ycf3*	*ycf4*			
	Photosystem II	19	*psbA*	*psbB*	*psbC*	*psbD*	*psbE*	*psbF*	*psbH*	*psbI*
			*psbJ*	*psbK*	*psbL*	*psbN*	*psbT*	*psbV*		
			*psbW*	*psbX*	*psbY*	*psbZ*	*psb30*			
	Cytochrome complex	11	*ccs1*	*ccsA*	*petA*	*petB*	*petD*	*petF*	*petG*	*petJ*
			*petL*	*petM*	*petN*					
	Redox system	7	*acsF*	*bas1*	*dsbD*	*ftrB*	*grx*	*pbsA*	*trxA*	
**ATP Synthesis**	ATP synthase	8	*atpA*	*atpB*	*atpD*	*atpE*	*atpF*	*atpG*	*atpH*	*atpI*
**Metabolism**	Carbohydrates	6	*cfxQ*	*pdhA*	*pdhB*	*pgmA*	*rbcL*	*rbcS*		
	Lipids	5	*accA*	*accB*	*accD*	*acpP*	*fabH*			
	Nucleotides	2	*carA*	*upp*						
	Amino acids (AAs)	8	*argB*	*gltB*	*ilvB*	*ilvH*	*hisS*	*syfB*	*trpA*	*trpG*
	Cofactors	8	*chlB*	*chlI*	*chlL*	*chlN*	*moeB*	*preA*	*thiG*	*thiS*
	Secondary metabolites	1	*dfr*							
**Transport**	Transport	9	*cemA*	*secA*	*secG*	*secY*	*sufB*	*sufC*	*tatC*	*ycf38*
			*ycf63*							
**Unknown**	Conserved open reding frame (ORF)s	23	*ycf19*	*ycf20*	*ycf21*	*ycf22*	*ycf23*	*ycf33*	*ycf34*	*ycf35*
			*ycf36*	*ycf37*	*ycf39*	*ycf41*	*ycf45*	*ycf46*	*ycf52*	*ycf53*
			*ycf54*	*ycf55*	*ycf56*	*ycf60*	*ycf65*	*ycf80*	*ycf92*	
	Unique ORFs	6	*orf55*	*orf88*	*orf169*	*orf402*	*orf441*	*orf768*		
Total genes		205								

**Table 2:** Comparison of general plastid structure in red algae similar to *Palmaria* sp. (Japan).

Subclass	Species	General Characteristics				RNAs		GenBank Accession	Reference
		Total nt	GC% *	Introns	PCG *^2^	tRNA	rRNA		
Nemaliophycidae	*Palmaria* sp. (Japan)	192,410	34.6	2	205	33	6	AB807662	This study
	*Palmaria palmata*	192,960	33.9	2	205	33	6	NC_031147	[25]
	*Kumanoa americana hys120*	184,025	29.3	2	201	32	3	NC_031178	[25]
	*Thorea hispida hsy077*	175,193	28.3	2	194	31	3	NC_031171	[25]
Corallinophycidae	*Calliarthron tuberculosum*	178,981	29.2	2	202	33	3	NC_021075	[26]
	*Sporolithon durum*	191,464	29.3	2	207	30	3	NC_029857	[27]
Ahnfeltiophycidae	*Ahnfeltia plicata*	190,451	32.5	1	207	31	6	NC_031145	[28]
Rhodymeniophycidae	*Asparagopsis taxiformis*	177,091	29.4	2	205	32	3	NC_031148	[28]
	*Ceramium japonicum*	171,634	27.8	1	202	29	3	NC_031174	[28]
	*Rhodymenia pseudopalmata*	194,153	32.0	1	202	32	6	NC_031144	[28]
	*Vertebrata lanosa*	167,158	30.0	0	193	28	3	KP308097	[29]

* A percentage of guanine and cytosine in a plastid genome DNA; *^2^ protein coding genes.

**Table 3:** Composition of AAs in *Palmaria* sp. (Japan) plastid protein and AAs in *P. palmata.*

AA	Plastid	GS	RP	PS	ATP	Meta	TP	UK	*P. palmata*	
	% of AA								% of Total AA ^a^ or Protein ^b^	
Alanine	6.4	5.6	6.8	7.6	8.7	6.4	5.8	4.5	7.5 ^a^	6.7 ^b^
Arginine	4.6	5.1	6.8	4.1	3.8	4.0	3.6	4.1	6.2 ^a^	5.1 ^b^
Aspartic acid	4.5	5.6	4.1	4.0	4.6	5.4	3.9	3.8	9.3 ^a^	18.5 ^b^
Asparagine	5.5	5.9	5.2	4.8	4.2	5.6	5.6	6.4		
Cystine	1.1	0.8	0.7	1.0	0.2	1.4	1.2	1.5	1.3 ^a^	0 ^b^
Glutamic acid	5.7	6.4	6.2	4.6	7.2	6.0	6.2	4.7	13 ^a^	9.9 ^b^
Glutamine	4.2	4.5	4.1	3.8	5.5	4.3	3.9	4.3		
Glycine	6.3	5.7	7.0	7.6	7.1	6.6	5.4	3.7	7.2 ^a^	13.3 ^b^
Histidine	1.9	1.9	1.9	1.9	0.7	2.3	1.4	2.1	2.1 ^a^	0.5 ^b^
Isoleucine	9.0	10.0	8.7	7.5	9.3	9.0	11.1	10.0	5.3 ^a^	3.7 ^b^
Leucine	10.6	10.1	8.7	10.4	12.0	10.4	12.2	12.9	7.8 ^a^	7.1 ^b^
Lysine	6.5	7.3	9.3	4.4	5.5	5.8	5.3	7.0	8.2 ^a^	3.3 ^b^
Methionine	2.2	1.8	2.1	2.6	2.0	2.4	1.9	1.8	1.9 ^a^	2.7 ^b^
Phenylalanine	4.1	3.2	2.7	5.8	3.4	3.5	4.8	5.1	5.2 ^a^	5.1 ^b^
Proline	3.7	3.6	3.6	4.1	3.7	3.9	3.1	3.4	4.4 ^a^	
Serine	7.4	7.0	6.3	7.8	6.8	7.2	7.8	8.6	4.6 ^a^	6.3 ^b^
Threonine	5.6	5.2	5.8	5.6	5.9	5.6	6.0	5.3	4.5 ^a^	3.6 ^b^
Tryptophan	1.0	0.5	0.5	1.9	0.4	0.8	0.9	1.3		
Tyrosine	3.6	3.5	2.6	3.9	2.4	3.4	4.2	4.7	4.5 ^a^	3.4 ^b^
Valine	6.3	6.6	7.0	6.6	6.9	6.2	5.9	4.9	7.3 ^a^	6.9 ^b^
Total AA	50,333	7010	8981	11,017	1975	11,213	3184	6953		

^a^ [6]; ^b^ [31]. GS: genetic system; RB: ribosomal proteins; PS: photosystems; ATP: ATP synthesis; Meta: metabolism; TP: transport; UK: unknown.

**Table 4:** Angiotensin I converting enzyme (ACE) inhibitory peptides from *Palmaria* sp. (Japan) plastid.

Peptide *	Database *^2^	Plastid	GS	RP	PS	ATP	Meta	TP	UK
XXP	34	260	48	38	62	10	61	20	21
XXY	20	140	21	13	30	7	31	13	25
XXA	6	66	9	11	21	5	13	3	4
XXL	5	78	5	11	27	2	19	5	9
XXW	5	4	0	0	3	0	1	0	0
XXG	3	51	5	16	8	0	16	2	4
XXR	3	31	4	10	3	0	7	0	7
XXV	3	33	5	8	10	1	6	2	1
XXF	2	7	0	0	3	0	2	0	2
XXK	2	39	8	12	3	3	8	1	4
XXN	2	5	0	1	1	0	2	0	1
XXX *^3^	4	39	8	8	6	0	10	5	2
Total	89	751	113	128	177	28	176	51	80
Total AA		50,333	7010	8981	11,017	1975	11,213	3184	6953
Peptide/AA (%)		1.49	1.61	1.43	1.61	1.42	1.57	1.60	1.15

* The peptide structures and related proteins are listed in Table S2; *^2^ No. of peptides having IC_50_ (>20 μM) are obtained from BIO-PEP-UWM database; *^3^ Four tripeptide sequences: LVQ, LVE, IWH, GPM; GS: genetic system; RB: ribosomal proteins; PS: photosystems; ATP: ATP synthesis; Meta: metabolism; TP: transport; UK: unknown.

**Table 5:** ACE inhibitory peptides from photosystems.

Peptide *	PBS	PSI	PSII	Cc	Red
XXP	11	21	17	7	6
XXY	9	8	7	3	3
XXA	6	4	8	1	2
XXL	6	12	4	5	0
XXW	0	3	0	0	0
XXG	3	1	4	0	0
XXR	1	1	1	0	0
XXV	3	2	3	2	0
XXF	0	0	2	1	0
XXK	0	1	2	0	0
XXN	0	0	0	0	1
XXX **	3	0	3	0	0
Total	42	53	51	19	12
Total AA	2644	2654	2582	1784	1353
Peptide/AA (%)	1.59	2.00	1.98	1.07	0.89

* The peptide structures and related proteins are listed in Table S3; ** LVQ, LVE, IWH, GPM; PS: photosystems; PBS: phycobilisomes; PSI: photosystem I; PSII: photosystem II; Cc: cytochrome complex; Red: redox system.

**Table 6:** ACE inhibitory peptide in photosystem proteins.

PBS	No. *	PSI	No. *	PSII	No. *	PSII	No. *	Cc	No. *	Red	No. *
apcA	2	psaA	18	psbA	9	psbV	2	ccs1	5	acsF	2
apcB	2	psaB	13	psbB	11	psbW	1	ccsA	0	bas1	1
apcD	3	psaC	1	psbC	10	psbX	0	petA	5	dsbD	4
apcE	14	psaD	1	psbD	6	psbY	1	petB	3	ftrB	1
apcF	3	psaE	2	psbE	1	psbZ	2	petD	1	grx	0
cpcG	0	psaF	5	psbF	0	psb30	0	petF	3	pbsA	2
cpcS	2	psaI	1	psbH	3			petG	1	trxA	2
rpcA	2	psaJ	0	psbI	0			petJ	0		
rpcB	3	psaL	3	psbJ	1			petL	0		
rpeA	6	psaM	1	psbK	1			petM	1		
rpeB	4	psbH	1	psbL	1			petN	0		
nblA	1	ycf3	3	psbN	0						
		ycf4	4	psbT	2						
Total	42		53				51		19		12

* No. of ACE inhibitory peptides; PBS: phycobilisomes; PSI: photosystem I; PSII: photosystem II; Cc: cytochrome complex; Red: redox system.

**Table 7:** ACE inhibitory peptides from *P. palmata* plastid.

Peptide *	Database *^2^	Plastid	GS	RP	PS	ATP	Meta	TP	UK
XXP	34	263	47	39	63	10	63	20	21
XXY	20	133	23	13	29	7	28	12	21
XXA	6	68	8	13	20	6	13	3	5
XXL	5	79	6	10	25	2	20	5	11
XXW	5	4	0	0	3	0	1	0	0
XXG	3	51	6	16	8	0	16	2	3
XXR	3	27	2	10	3	0	7	0	5
XXV	3	33	5	8	9	1	7	2	1
XXF	2	6	0	0	3	0	2	0	1
XXK	2	37	9	11	3	4	7	1	2
XXN	2	5	0	1	1	0	2	0	1
XXX *^3^	4	36	7	8	6	0	9	5	1
Total	89	742	113	129	173	30	175	50	72
Total AA		50,229	7009	8981	11,013	1970	11,237	3188	6831
Peptide/AA (%)		1.48	1.61	1.44	1.57	1.52	1.56	1.57	1.05

* The peptide structures and related proteins are listed in Table S4; *^2^ No. of peptides having IC_50_ (>20 μM) were obtained from the BIO-PEP-UWM database; *^3^ LVQ, LVE, IWH, GPM; GS: genetic system; RB: ribosomal proteins; PS: photosystems; ATP: ATP synthesis; Meta: metabolism; TP: transport; UK: unknown.

## Supplementary Materials

The following are available online at https://www.mdpi.com/1660-3397/17/3/190/s1, Table S1: Primers for *Palmaria* sp. (Japan) plastid sequence, Table S2: ACE inhibitory peptide from *Palmaria* sp. (Japan) plastid, Table S3: ACE inhibitory peptide from *Palmaria* sp. (Japan) photosystems, Table S4: ACE inhibitory peptide from *Palmaria palmata* plastid.
